# Risk factors for extended length of stay and non-home discharge in adults treated with multi-level fusion for lumbar degenerative pathology and deformity

**DOI:** 10.1007/s43390-022-00620-7

**Published:** 2022-12-15

**Authors:** Ayush Arora, Aboubacar Wague, Ravi Srinivas, Matt Callahan, Thomas A. Peterson, Alekos A. Theologis, Sigurd Berven

**Affiliations:** 1 Department of Orthopaedic Surgery, University of California - San Francisco (UCSF), San Francisco, 500 Parnassus Ave, MUW320W, San Francisco, CA USA; 2 Bakar Computational Health Sciences Institute, UCSF, San Francisco, CA USA

**Keywords:** Adult spinal deformity, Lumbar degenerative pathology, Preoperative optimization, Social support, Risk stratification, Surgical invasiveness

## Abstract

**Purpose:**

To identify independent risk factors, including the Risk Assessment and Prediction Tool (RAPT) score, associated with extended length of stay (eLOS) and non-home discharge following elective multi-level instrumented spine fusion operations for diagnosis of adult spinal deformity (ASD) and lumbar degenerative pathology.

**Methods:**

Adults who underwent multi-level ($\ge 3$ segments) instrumented spine fusions for ASD and lumbar degenerative pathology at a single institution (2016–2021) were reviewed. Presence of a pre-operative RAPT score was used as an inclusion criterion. Excluded were patients who underwent non-elective operations, revisions, operations for trauma, malignancy, and/or infections. Outcomes were eLOS (> 7 days) and discharge location (home vs. non-home). Predictor variables included demographics, comorbidities, operative information, Surgical Invasiveness Index (SII), and RAPT score. Fisher’s exact test was used for univariate analysis, and significant variables were implemented in multivariate binary logistic regression, with generation of 95% percent confidence intervals (CI), odds ratios (OR), and p-values.

**Results:**

Included for analysis were 355 patients. Post-operatively, 36.6% (*n *= 130) had eLOS and 53.2% (*n* = 189) had a non-home discharge. Risk factors significant for a non-home discharge were older age (> 70 years), SII > 36, pre-op RAPT < 10, DMII, diagnosis of depression or anxiety, and eLOS. Risk factors significant for an eLOS were SII > 20, RAPT < 6, and an ASA score of 3.

**Conclusion:**

The RAPT score and SII were most important significant predictors of eLOS and non-home discharges following multi-level instrumented fusions for lumbar spinal pathology and deformity. Preoperative optimization of the RAPT’s individual components may provide a useful strategy for decreasing LOS and modifying discharge disposition.

## Introduction

Adult spinal deformity (ASD) is a debilitating condition with detrimental impact on health-related quality of life (HRQOL). Metrics, including the Owestry Disability Index (ODI), Short-Form 36 (SF-36), and Scoliosis Research Society score (SRS), have demonstrated that ASD patients have greater disability than patients with other chronic conditions [1–4].

Surgical intervention for ASD patients often necessitates considerable time for recovery in the hospital and after discharge. Extended length of stay and discharge to a rehabilitation center following operations for multi-level instrumented spinal fusions are important outcomes that are significantly associated with complications and higher costs [5, 6]. eLOS is notable as a composite determination of the postoperative course, as patients with elderly age, systemic illnesses, and medical complications stay longer in the hospital due to need for extended management. Discharge to a rehabilitation or skilled nursing facility (SNF) is a commonly pursued postoperative plan for patients requiring aid in return to function and extended acute care, accounting for at least 30% of the total cost of care [7].

There is thus a need to accurately identify risk factors pre-operatively associated with eLOS and non-home discharges for patients undergoing multi-level fusions for ASD and lumbar degenerative pathology. Preoperative identification of such patients is important for financial risk minimization, patient informed choice, preemptive administration action, and better shared decision-making ability [8–10]. The current data on risk factors for patients undergoing surgery for ASD require additional granularity. Few studies adequately stratify patients, often utilizing large-administrative databases without sufficiently discriminating between patient specific diagnosis and procedure [11, 12]. Additionally, few studies in spine literature utilize social support, a predictive variable for complications that is extensively validated in other surgical specialties [13, 14]. One tool used to capture social support is the Risk Assessment and Prediction Tool (RAPT). The RAPT score (Table 1) holds significant potential in predicting patient outcomes following surgery as a cumulative scaled score ranging from 1 to 12, comprised of components that correspond to patient community support, extent of home care, gait aid, and preoperative functional ability [15, 16].

**Table 1:** Risk Assessment and Prediction Tool (RAPT) Questionnaire

Question	Value	Score
1. What is your age group?	50–65 years 66–75 years > 75 years	= 2 = 1 = 0
2. Gender?	Male Female	= 2 = 1
3. How far, on average, can you walk? (a block is 200 m)	Two blocks or more (± rests) 1–2 blocks (the shopping center) Housebound (most of the time)	= 2 = 1 = 0
4. Which gait aid do you use? (more often than not)	None Single point stick Crutches/frame	= 2 = 1 = 0
5. Do you use community supports? (home help, meals-on wheels, district nurse)	None or one per week Two or more per week	= 1 = 0
6. Will you live with someone who can care for you after your operation?	Yes No	= 3 = 0
Total score (out of 12)		

The purpose of this study is to identify independent risk factors, including the RAPT score, associated with eLOS and non-home discharge following elective multi-level operations for ASD and lumbar degenerative pathology.

## Methods

### Data source

Following Institutional Review Board approval, records for patients undergoing elective surgery for ASD and lumbar degenerative pathology from March 1, 2016 to October 1, 2021 at a single academic tertiary care center were obtained retrospectively from the Electronic Health Record (EHR). Data acquired from patient charts included demographic information, operative details, surgical invasiveness index (SII), comorbidities, and pre-operative RAPT score.

### Inclusion and exclusion criteria

Eligibility criteria consisted of adult patients (ages ≥ 50 years) with a diagnosis of ASD or lumbar degenerative pathology (i.e. idiopathic/degenerative scoliosis, flatback deformity, spinal stenosis, spondylosis, spondylolisthesis, degenerative disc disease) undergoing elective multi-level fusions with or without decompressions in the lumbar or thoracolumbar spine. Multi-level was defined as ≥ 3 segments. Presence of a pre-operative RAPT score was also used as an inclusion criterion. Excluded were those patients with incomplete data, underwent non-elective operations, revisions, operations for trauma, malignancy, and/or infections. Patients with unknown discharge location, discharge against medical advice, and/or death were also excluded. Inclusion and exclusion criteria were derived from information listed on the patient’s operative note, procedure description, and demographic information listed on the patient chart.

### Risk factor variables

Predictor variables included demographics, comorbidities, operative information, and social support. Demographics obtained for each patient included age (years: 50–59, 60–69, 70–79, ≥ 80), gender (male vs. female), race (Asian, White, Black, Native American/Pacific Islander, Multiracial), and body mass index (BMI; normal, overweight, obese, morbidly obese). Operative information included surgical invasiveness index (SII), estimated blood loss (EBL: < 50 mL, 50–99 mL, 100–199 mL, 200–299 mL, ≥ 300 mL), and American Society of Anesthesiologists Score (1–2, 3). Of note, SII is an extensively validated tool developed by *Mirza *et al*.*, measured by adding the number of vertebral levels receiving decompression, fusion, and/or instrumentation from the anterior and posterior approaches [17]. The SII has a score range of 0–48 and was manually calculated for each patient based on the textual information in the procedure operative note. Social support was quantified by the RAPT score (poor 0–5, moderate: 6–9, good 10–12) [16]. Other recorded comorbidities included substance use disorders (alcohol, illicit drugs, preoperative opioid use), smoking history (never smoker, former smoker, current smoker), Charlson Comorbidity Index (CCI 0–1, 2–3, 4 +), and individual medical conditions [congestive heart failure (CHF), hypertension (HTN), chronic obstructive pulmonary disease (COPD), diabetes (DMII), chronic kidney disease (CKD), inflammatory disease, osteoporosis, depression, anxiety). Patient comorbidities were acquired from algorithms incorporating International Classification of Diseases, Volumes 10 codes (ICD10) [18].

### Outcome variables

The two primary outcomes were eLOS (> 7 days) and discharge outcome (home vs non-home). A non-home discharge was defined as a patient transfer to a skilled nursing facility (SNF) or post-acute rehabilitation facility following surgery.

### Statistical analysis

Fisher’s exact test was used for univariate analysis to determine risk factors that were significant for eLOS and discharge disposition. Significant variables were then implemented in multivariate analysis via binary logistic regression, with generation of 95% percent confidence intervals (CI), odds ratios (OR), and *p*-values for each respective risk factor. A *p*-value < 0.05 was deemed statistically significant. All statistical tests were conducted using MATLAB 2021b statistical toolbox [19].

## Results

### Exploratory data analysis

Included for analysis were 355 patients. Of these, 36.6% had an eLOS (Table 2) and 53.2% had a non-home discharge (Table 3). Of patients with eLOS, 27.7% were discharged to home and 72.3% were discharged to a non-home location. Of patients with non-eLOS( ≤ 7 days), 57.8% were discharged to home and 42.2% were discharged to a non-home location. The cohort had a male to female distribution of 37.5% and 62.5%, respectively. Procedures were heterogenous, as indicated by the variation in SII: with 17.7% (SSI: 5–15), 20.6% (SSI: 16–20), 12.4% (SSI: 21–25), 15.2% (SSI: 26–30), 17.5% (SSI: 31–35), and 11.3% (SSI: ≥ 36). The majority of patients had a RAPT score (6–9) that corresponded to moderate risk (52.4%). Common comorbidities were HTN (54.9%) and DMII (22.8%) (Tables 2 and 3).

**Table 2:** Baseline data of patients who underwent elective long-segment operations for lumbar degenerative pathology and deformity, stratified by length of stay

Variable	N (% of cohort)	Length of stay ≤ 7 days (%)	Length of stay > 7 Days (%)
Population	355 (100.0%)	225 (63.4%)	130 (36.6%)
Age (Mean, SD)	66.9 ± 8.0	66.1 ± 7.7	68.3 ± 8.1
50–59	73 (20.6%)	52 (71.2%)	21 (28.8%)
60–69	141 (39.7%)	96 (68.1%)	45 (31.9%)
70–79	122 (34.4%)	68 (55.7%)	54 (44.3%)
≥ 8	19 (5.4%)	9 (47.4%)	10 (52.6%)
Gender			
Male	133 (37.5%)	80 (60.2%)	53 (39.8%)
Female	222 (62.5%)	145 (65.3%)	77 (34.7%)
Race			
Asian	17 (4.8%)	14 (82.4%)	3 (17.6%)
White	284 (80.0%)	172 (60.6%)	112 (39.4%)
Black	16 (4.5%)	14 (87.5%)	2 (12.5%)
Hispanic	18 (5.1%)	9 (50.0%)	9 (50.0%)
Native American/Pacific Islander	6 (1.7%)	3 (50.0%)	3 (50.0%)
Multiracial	12 (3.4%)	11 (91.7%)	1 (8.3%)
Declined to State	2 (0.6%)	2 (100.0%)	0 (0.0%)
BMI			
Normal BMI (BMI < 25)	126 (35.5%)	81 (64.3%)	45 (35.7%)
Overweight (25 ≤ BMI < 30)	110 (31.0%)	72 (65.5%)	38 (34.5%)
Obese (30 ≤ BMI < 35)	66 (18.6%)	42 (63.6%)	24 (36.4%)
Morbidly obese (BMI ≥ 35)	53 (14.9%)	30 (56.6%)	23 (43.4%)
Approach			
Posterior	92 (25.9%)	68 (73.9%)	24 (26.1%)
Anterior and posterior fusion	263 (74.1%)	157 (59.7%)	106 (40.3%)
Surgical invasiveness index			
5–15	63 (17.7%)	61 (96.8%)	2 (3.2%)
16–20	73 (20.6%)	60 (82.2%)	13 (17.8%)
21–25	44 (12.4%)	29 (65.9%)	15 (34.1%)
26–30	54 (15.2%)	29 (53.7%)	25 (46.3%)
31–35	62 (17.5%)	25 (40.3%)	37 (59.7%)
≥ 36	40 (11.3%)	10 (25.0%)	30 (75.0%)
Estimated blood loss			
< 50	25 (7.0%)	21 (84.0%)	4 (16.0%)
50–99	47 (13.2%)	29 (61.7%)	18 (38.3%)
100–199	36 (10.1%)	22 (61.1%)	14 (38.9%)
200–299	45 (12.7%)	34 (75.6%)	11 (24.4%)
≥ 300	202 (56.9%)	119 (58.9%)	83 (41.1%)
ASA			
1–2	203 (57.2%)	141 (69.5%)	62 (30.5%)
3	149 (42.0%)	82 (55.0%)	67 (45.0%)
4	4 (1.1%)	4 (100.0%)	0 (0.0%)
RAPT			
0–5	51 (14.4%)	23 (45.1%)	28 (54.9%)
6–9	186 (52.4%)	114 (61.3%)	72 (38.7%)
10–12	118 (33.2%)	88 (74.6%)	30 (25.4%)
Substance use			
Alcohol	170 (47.9%)	106 (62.4%)	64 (37.6%)
Illicit drugs	66 (18.6%)	45 (68.2%)	21 (31.8%)
Preoperative opioid use	212 (59.7%)	132 (62.3%)	80 (37.7%)
Smoking history			
Never smoker	186 (52.4%)	118 (63.4%)	68 (36.6%)
Former smoker	131 (36.9%)	81 (61.8%)	50 (38.2%)
Current smoker	38 (10.7%)	26 (68.4%)	12 (31.6%)
Charlson comorbidity index			
0–1	50 (14.1%)	37 (74.0%)	13 (26.0%)
2–3	193 (54.4%)	124 (64.2%)	69 (35.8%)
4 +	112 (31.5%)	64 (57.1%)	48 (42.9%)
Comorbidities			
CHF	13 (3.7%)	6 (46.2%)	7 (53.8%)
HTN	195 (54.9%)	118 (60.5%)	77 (39.5%)
COPD	72 (20.3%)	42 (58.3%)	30 (41.7%)
DMII	81 (22.8%)	52 (64.2%)	29 (35.8%)
CKD	29 (8.2%)	16 (55.2%)	13 (44.8%)
Inflammatory disease	21 (5.9%)	15 (71.4%)	6 (28.6%)
Osteoporosis	58 (16.3%)	31 (53.4%)	27 (46.6%)
Depression/anxiety	78 (22.0%)	43 (55.1%)	35 (44.9%)

*BMI* body mass index, *RAPT* risk assessment and prediction tool, *CHF* congestive heart failure, *HTN* hypertension, *COPD* chronic obstructive pulmonary disease, *DMII* diabetes mellitus type II, *CKD* chronic kidney disease, *SD* standard deviation

**Table 3:** Baseline data of patients who underwent elective long-segment operations for lumbar degenerative pathology and deformity, stratified by discharge disposition

Variable	N (% of cohort)	Home discharge (%)	Non-home discharge (%)
Population	355 (100.0%)	166 (46.8%)	189 (53.2%)
Age (Mean, SD)	66.9 ± 8.0	63.7 ± 7.1	69.7 ± 7.6
50–59	73 (20.6%)	50 (68.5%)	23 (31.5%)
60–69	141 (39.7%)	79 (56.0%)	62 (44.0%)
70–79	122 (34.4%)	35 (28.7%)	87 (71.3%)
≥ 80	19 (5.4%)	2 (10.5%)	17 (89.5%)
Gender			
Male	133 (37.5%)	68 (51.1%)	65 (48.9%)
Female	222 (62.5%)	98 (44.1%)	124 (55.9%)
Race			
Asian	17 (4.8%)	7 (41.2%)	10 (58.8%)
White	284 (80.0%)	133 (46.8%)	151 (53.2%)
Black	16 (4.5%)	7 (43.8%)	9 (56.3%)
Hispanic	18 (5.1%)	7 (38.9%)	11 (61.1%)
Native American/Pacific Islander	6 (1.7%)	4 (66.7%)	2 (33.3%)
Multiracial	12 (3.4%)	7 (58.3%)	5 (41.7%)
Declined to State	2 (0.6%)	1 (50.0%)	1 (50.0%)
BMI			
Normal BMI (BMI < 25)	126 (35.5%)	66 (52.4%)	60 (47.6%)
Overweight (25 ≤ BMI < 30)	110 (31.0%)	50 (45.5%)	60 (54.5%)
Obese (30 ≤ BMI < 35)	66 (18.6%)	28 (42.4%)	38 (57.6%)
Morbidly Obese (BMI ≥ 35)	53 (14.9%)	22 (41.5%)	31 (58.5%)
Approach			
Posterior	92 (25.9%)	49 (53.3%)	43 (46.7%)
Anterior and Posterior	263 (74.1%)	117 (44.5%)	146 (55.5%)
Surgical invasiveness index			
5–15	63 (17.7%)	43 (68.3%)	20 (31.7%)
16–20	73 (20.6%)	39 (53.4%)	34 (46.6%)
21–25	44 (12.4%)	25 (56.8%)	19 (43.2%)
26–30	54 (15.2%)	21 (38.9%)	33 (61.1%)
31–35	62 (17.5%)	20 (32.3%)	42 (67.7%)
≥ 36	40 (11.3%)	8 (20.0%)	32 (80.0%)
Estimated blood loss			
< 50	25 (7.0%)	11 (44.0%)	4 (56.0%)
50–99	47 (13.2%)	22 (46.8%)	25 (53.2%)
100–199	36 (10.1%)	19 (52.8%)	17 (47.2%)
200–299	45 (12.7%)	29 (64.4%)	16 (35.6%)
≥ 300	202 (56.9%)	85 (42.1%)	11 (57.9%)
ASA			
1–2	203 (57.2%)	106 (52.2%)	97 (47.8%)
3	149 (42.0%)	59 (39.6%)	90 (60.4%)
4	4 (1.1%)	0 (0.0%)	4 (100.0%)
RAPT			
0–5	51 (14.4%)	6 (11.8%)	45 (88.2%)
6–9	186 (52.4%)	73 (39.2%)	113 (60.8%)
10–12	118 (33.2%)	87 (73.7%)	31 (26.3%)
Substance use			
Alcohol	170 (47.9%)	82 (48.2%)	88 (51.8%)
Illicit drugs	66 (18.6%)	55 (42.0%)	28 (42.4%)
Preoperative Opioid Use	212 (59.7%)	20(52.6%)	115 (54.2%)
Smoking history			
Never Smoker	186 (52.4%)	91 (48.9%)	95 (51.1%)
Former Smoker	131 (36.9%)	55 (42.0%)	76 (58.0%)
Current Smoker	38 (10.7%)	20 (52.6%)	18 (47.4%)
Charlson comorbidity index (CCI)			
0–1	50 (14.1%)	37 (74.0%)	13 (26.0%)
2–3	193 (54.4%)	102 (52.8%)	91 (47.2%)
4 +	112 (31.5%)	27 (24.1%)	85 (75.9%)
Comorbidities			
CHF	13 (3.7%)	3 (23.1%)	10 (76.9%)
HTN	195 (54.9%)	85 (43.6%)	110 (56.4%)
COPD	72 (20.3%)	35 (48.6%)	37 (51.4%)
DMII	81 (22.8%)	26 (32.1%)	55 (67.9%)
CKD	29 (8.2%)	6 (20.7%)	23 (79.3%)
Inflammatory disease	21 (5.9%)	10 (47.6%)	11 (52.4%)
Osteoporosis	58 (16.3%)	20 (34.5%)	38 (65.5%)
Depression/anxiety	78 (22.0%)	24 (30.8%)	54 (69.2%)
Length of stay			
Non-eLOS	225 (63.4%)	130 (57.8%)	95 (42.2%)
eLOS	130 (36.6%)	36 (27.7%)	94 (72.3%)

*BMI* body mass index, *ASA* American anesthesiologist score, *RAPT* risk assessment and prediction tool, *CHF* congestive heart failure, *HTN* hypertension, *COPD* chronic obstructive pulmonary disease, *DMII* diabetes mellitus type II, *CKD* chronic kidney disease, *SD* standard deviation

### Statistical analysis: extended length of stay

Univariate and multivariate analyses for eLOS are displayed in Table 4. Following multivariate analysis, variables that had a significant positive association with eLOS included: SII between 21 and 25 (OR = 3.58, 95% CI 1.58–8.13, *p* = 0.002), SII between 26 and 30 (OR = 4.91, 95% CI 2.31–10.44, *p* < 0.001), SII between 31 and 35 (OR = 6.19, CI 2.99–12.80, *p* < 0.001), SII ≥ 36 (OR = 16.44, CI 6.81–39.70, *p* < 0.001), ASA score of three (OR = 2.05, CI 1.21–3.49, *p* = 0.008), and RAPT score of 0–5 (OR = 3.15, 95% CI 1.37–7.20, *p* = 0.007) (Table 4).

**Table 4:** Univariate and multivariate analyses of significant predictors for extended length of stay

Variable	Univariate tests			Multivariate tests		
Population	OR	95% CI	P	OR	95% CI	P
Age						
50–59	*Ref*	–	–	–	–	–
60–69	1.16	0.63–2.15	0.755	–	–	–
70–79	1.97	1.06–3.66	0.034	0.99	0.57–1.72	0.984
≥ 80	2.75	0.98–7.73	0.061	–	–	–
Gender						
Female	*Ref*	–	–	–	–	–
Male	1.25	0.8–1.94	0.363	–	–	–
Race						
White	*Ref*	–	–	–	–	–
Asian	0.33	0.09–1.17	0.12	–	–	–
Black	0.22	0.05–0.98	0.034	0.59	0.12–2.83	0.506
Hispanic	1.54	0.59–3.99	0.459	–	–	–
Native American/Pacific islander	1.54	0.3–7.74	0.684	–	–	–
Multiracial	0.14	0.02–1.1	0.034	–	–	–
BMI						
Normal BMI (BMI < 25)	*Ref*	–	–	–	–	–
Overweight (25 ≤ BMI < 30)	1.01	0.57–1.78	1	–	–	–
Obese (30 ≤ BMI < 35)	1.03	0.56–1.87	1	–	–	–
Morbidly obese (BMI ≥ 35)	1.29	0.66–2.53	0.494	–	–	–
Approach						
Posterior	*Ref*					
Anterior and posterior	1.91	1.13–3.24	0.017	1.8	0.92–3.5	0.084
Surgical invasiveness index						
May-15	*Ref*	–	–	–	–	–
16–20	6.61	1.43–30.54	0.011	–	–	0.002
21–25	15.78	3.38–73.6	< 0.001	3.58	1.58–8.13	< 0.001
26–30	26.29	5.83–118.62	< 0.001	4.91	2.31–10.44	< 0.001
31–35	45.14	10.1–201.71	< 0.001	6.19	2.99–12.8	< 0.001
≥ 36	55.19	12.24–248.81	< 0.001	16.44	6.81–39.7	
Estimated blood loss						
< 50	*Ref*	–	–	–	–	–
50–99	3.26	0.96–11.04	0.063	–	–	–
100–199	3.34	0.95–11.8	0.086	–	–	–
200–299	1.7	0.48–6.03	0.548	–	–	–
≥ 300	3.66	1.21–11.06	0.016	1.61	0.94–2.75	0.084
ASA						
01-Feb	*Ref*	–	–	–	–	–
3	1.86	1.2–2.88	0.007	2.05	1.21–3.49	0.008
RAPT						
10-Dec	*Ref*	–	–	–	–	–
06-Sep	1.85	1.11–3.08	0.018	1.48	0.79–2.79	0.225
0–5	3.57	1.79–7.12	< 0.001	3.15	1.37–7.2	0.007
Substance use						
Alcohol	0.97	0.58–1.6	0.899	–	–	–
Illicit drugs	0.77	0.44–1.36	0.399	–	–	–
Preoperative opioid use	12.39	2.75–55.82	< 0.001	0.99	0.58–1.68	0.962
Smoking history						
Never smoker	*Ref*	–	–	–	–	–
Former smoker	1.07	0.67–1.7	0.814	–	–	–
Current smoker	0.8	0.38–1.69	0.711	–	–	–
Charlson comorbidity index						
0–1	*Ref*	–	–	–	–	–
02-Mar	1.58	0.79–3.18	0.128	–	–	–
4 +	1.33	0.62–2.87	0.567	–	–	–
Comorbidities						
CHF	2.08	0.68–6.32	0.242	–	–	–
HTN	1.32	0.85–2.04	0.225	–	–	–
COPD	1.31	0.77–2.22	0.339	–	–	–
DMII	0.98	0.58–1.64	1	–	–	–
CKD	1.45	0.67–3.12	0.421	–	–	–
Inflammatory disease	0.68	0.26–1.79	0.492	–	–	–
Osteoporosis	1.64	0.93–2.9	0.101	–	–	–
Depression/anxiety	1.56	0.94–2.6	0.11	–	–	–

*BMI* body mass index, *ASA* American Anesthesiologist Score, *RAPT* risk assessment and prediction tool, *CHF* congestive heart failure, *HTN* hypertension, *COPD* chronic obstructive pulmonary disease, *DMII* diabetes mellitus type II, *CKD* chronic kidney disease, *SD* standard deviation

### Statistical analysis: discharge location

Univariate and multivariate analyses for discharge disposition are displayed in Table 5. Following multivariate analysis, variables that had a significant positive association with non-home discharges included: age between 70 and 79 years (OR = 3.05, 95% CI 1.70–5.45, *p* = 0.002), age ≥ 80 (OR = 9.19, 95% CI 1.79–47.34, *p* = 0.008), SII ≥ 36 (OR = 5.08, CI 1.96–13.14, *p* < 0.001), RAPT Score of 6–9 (OR = 2.96, 95% CI 1.60–5.49, *p* = 0.001), RAPT score of 0–5 (OR = 10.76, 95% CI 3.77–30.69, *p* < 0.001), diagnosis of DMII (OR = 2.07, 95% CI 1.03–4.15, *p* = 0.041), diagnosis of depression or anxiety (OR = 2.05, 95% CI 1.06–3.98, *p* = 0.034) and eLOS (OR = 3.66, 95% CI 1.71–7.84, *p* < 0.001) (Table 5).

**Table 5:** Univariate and multivariate analyses of significant predictors of discharge to rehabilitation

Variable	Univariate tests			Multivariate tests		
Population	OR	95% CI	*P*-value	OR	95% CI	*p*-value
Age						
50–59	*Ref*	–	–	–	–	–
60–69	1.71	0.94–3.09	0.105	–	–	–
70–79	5.40	2.88–10.15	< 0.001	3.05	1.70–5.45	0.002
≥ 80	18.48	3.94–86.72	< 0.001	9.19	1.79–47.34	0.008
Gender						
Female	*Ref*	–	–	–	–	–
Male	0.76	0.49–1.16	0.227	–	–	–
Race						
White	*Ref*	–	–	–	–	–
Asian	1.26	0.47–3.40	0.804	–	–	–
Black	1.13	0.41–3.12	1.000	–	–	–
Hispanic	1.38	0.52–3.67	0.629	–	–	–
Native American/Pacific Islander	0.44	0.08–2.44	0.426	–	–	–
Multiracial	0.63	0.20–2.03	0.558	–	–	–
BMI						
Normal BMI(BMI < 25)	*Ref*	–	–	–	–	–
Overweight (25 ≤ BMI < 30)	1.52	0.88–2.61	0.134	–	–	–
Obese (30 ≤ BMI < 35)	0.75	0.39–1.45	0.412	–	–	–
Morbidly obese (BMI ≥ 35)	0.74	0.41–1.34	0.369	–	–	–
Approach						
Posterior	*Ref*	–	–	–	–	–
Anterior and posterior	1.42	0.88–2.29	0.182	–	–	–
Surgical invasiveness index						
5–15	*Ref*	–	–	–	–	–
16–20	1.87	0.93–3.78	0.083	–	–	–
21–25	1.63	0.74–3.36	0.307	–	–	–
26–30	3.38	1.58–7.24	0.002	1.23	0.58–2.60	0.583
31–35	4.52	2.13–9.57	< 0.001	1.63	0.78–3.43	0.195
≥ 36	4.90	2.27–10.55	< 0.001	5.08	1.96–13.14	0.001
Estimated blood loss						
< 50	*Ref*	–	–	–	–	–
50–99	0.89	0.34–2.37	1.000	–	–	–
100–199	0.70	0.25–1.96	0.605	–	–	–
200–299	0.43	0.16–1.18	0.132	–	–	–
≥ 300	1.08	0.47–2.50	1.000	–	–	–
ASA						
1–2	*Ref*	–	–	–	–	–
3	1.67	1.09–2.56	0.023	0.96	0.55–1.68	0.881
RAPT						
10–12	*Ref*					
6–9	4.34	2.62–7.20	< 0.001	2.96	1.60–5.49	0.001
0–5	21.05	8.18–54.17	< 0.001	10.76	3.77–30.69	< 0.001
Substance use						
Alcohol	0.50	0.30–0.83	0.008	1.00	0.59–1.70	0.992
Illicit drugs	1.33	0.87–2.02	0.201	–	–	–
Preoperative opioid use	6.06	1.35–27.25	0.008	0.84	0.48–1.47	0.550
Smoking history						
Never smoker	*Ref*	–	–	–	–	–
Former smoker	1.32	0.84–2.08	0.253	–	–	–
Current smoker	0.86	0.43–1.73	0.724	–	–	–
Charlson comorbidity index						
0–1	*Ref*	–	–	–	–	–
2–3	2.54	1.27–5.07	0.005	0.85	0.48–1.53	0595
4 +	2.32	0.99–5.41	0.060	–	–	–
Comorbidities						
CHF	3.04	0.82–11.22	0.095			
HTN	1.33	0.87–2.02	0.201			
COPD	0.91	0.54–1.53	0.792			
DMII	2.19	1.30–3.69	0.004	2.07	1.03–4.15	0.041
CKD	3.69	1.47–9.31	0.003	1.59	0.52–4.92	0.418
Inflammatory disease	0.96	0.40–2.33	1.000	–	–	–
Osteoporosis	1.84	1.02–3.31	0.045	0.90	0.43–1.91	0.787
Depression/anxiety	2.37	1.39–4.04	0.001	2.05	1.06–3.98	0.034
Length of stay						
Non-eLOS	*Ref*	–	–	–	–	–
eLOS	4.58	2.55–8.22	< 0.001	3.66	1.71–7.84	< 0.001

*BMI* body mass index, *ASA* American anesthesiologist score, *RAPT* risk assessment and prediction tool, *CHF* congestive heart failure, *HTN* hypertension, *COPD* chronic obstructive pulmonary disease, *DMII* diabetes mellitus type II, *CKD* chronic kidney disease, *SD* standard deviation

## Discussion

The goal of this study was to determine significant granular risk factors associated with eLOS (> 7 days) and non-home discharges (SNF or acute rehabilitation) for patients undergoing multi-level fusions (≥ 3 segments) for lumbar degenerative pathology and deformity. Risk factors significant for a non-home discharge were older age (> 70 years), SII > 36, pre-op RAPT < 10, DMII, diagnosis of depression or anxiety, and eLOS. The risk factors significant for an eLOS were SII > 20, RAPT < 6, and an ASA score of 3.

Higher surgical invasiveness, as determined by the SII, as well as lower pre-operative RAPT scores were the two most important predictors for eLOS and discharge location. The SII has been broadly accepted as a strong predictor of surgical site infection, operative time, and perioperative complications [20–22]. For every five-point jump in SII, the OR’s increased by greater than one, highlighting that the variable’s effects became more prominent at increased ranges. Regarding the significance of the RAPT variable, prior studies have verified low RAPT scores to be implicated with non-home discharges for deformity patients [23, 24]. However, this study is the first to explore and demonstrate the significant association between the pre-operative RAPT score and eLOS in patients undergoing multi-level instrumented spinal fusions.

The literature is concordant with our results of significant associations between other risk factors with eLOS and discharge location. For example, advanced age has been clearly implicated with non-home discharges for ASD patients, with most reports determining that ages > 60 confer elevated risk [25, 26]. Notably, advanced age was not associated with eLOS. One explanation for this finding is that administrative teams may plan transition to rehabilitation facilities well ahead of surgery for older patients, which in turn results in fewer discharge delays compared to younger patients who may unexpectedly require a non-home discharge. Additionally, while the association between BMI and complications in spine surgery is contested within the literature, our study showed that BMI was not significantly associated with either eLOS or discharge location [27, 28]. Diabetes mellitus type II has been reported as a significant predictor of non-home discharge in one study using an administrative database of ASD patients conducted by Abt. et al. [29]. Previous studies using metrics quantifying psychiatric distress [i.e. the Koenig Depression Scale (KDS)] and a preoperative diagnosis of depression have failed to find association with perioperative complications following multi-level fusions [30–32]. Therefore, our findings of significant association between depression/anxiety and eLOS are among the first reported for deformity patients [33]. Additionally, given that a subset of patients who have eLOS may inherently require post-acute care, it follows that the variable was significant for predicting rehabilitation discharge [34]. Similarly, higher ASA scores are representative of greater morbidity and mortality, and the literature verifies the ASA’s predictive validity for quantifying eLOS risk [35].

The major difference between this study’s findings and the current literature is that certain previously validated variables lacked significant association with either postoperative outcome on multivariate analysis. For example, elevated BMI, higher EBL, smoking history, higher CCI ranges, and osteoporosis have been significantly associated with worse postoperative outcomes in prior reports [36–38]. An explanation for this may be that granular data on procedure quantified in terms of SII, in addition to the RAPT score, override the importance of the other medical comorbidities within the multivariate analysis. Of note, EBL ranges of 200–299 mL produced a lower OR than 100–199 mL for both outcomes, though neither were significant. Prior literature implies that EBL may be overestimated in spine surgery, and our use of narrow EBL categorical ranges may have resulted in this discrepancy in ORs [39]. Furthermore, while current and former smokers were not significant for either outcome, the effect of smoking is typically more substantial in current smokers [40]. Quantification of cumulative number of cigarette packs smoked may present a better option in future studies.

Accurate preoperative assessment of risk for eLOS and discharge location for patients undergoing multi-level fusions has clinical utility and can enable substantial cost savings. Extended length of stay has been labeled as a significant predictor of catastrophic costs, defined as total cost of care > $100,000 [5]. *Boylan *et al*.* found that each additional day in the hospital for deformity patients incurs close to $5200 in hospital costs, with eLOS patients accruing at least $19,000 in additional hospital expenditures compared to shorter LOS counterparts [41]. The cohort size in this study (*N* = 94) who had both eLOS and a discharge to rehabilitation represent combined outcomes that are highly indicative of cost outliers, as the addition of rehabilitation can further account for at least 30% of the total cost of care [7].

In some cases, the outcomes of eLOS and discharge location are interdependent. Patients with an eLOS due to need for extended management may require rehabilitation to facilitate functional return [42]. The inverse statement also applies, as patients needing discharge to rehabilitation may stay longer in the hospital due to the lengthy referral process and administrative delays [7]. The interdependence between both outcomes was clearly displayed in our study, where 72.3% of the eLOS patients (*N* = 94) were discharged to rehabilitation, and eLOS was a significant predictor of discharge location. The implication is that patients at risk of one outcome also carry the corresponding risk of the other outcome, and therefore, represent large financial risk. The combined costs of eLOS and rehabilitation discharge may undermine the sustainability of surgical intervention, especially under bundled payment models [5]. Accurate preoperative identification of patients who will have an eLOS and/or rehabilitation discharge is the first step in mitigating financial risk and enabling medical centers to anticipate expected costs of care.

The results of this study can also inform the optimization of modifiable risk factors in efforts to influence a more favorable postoperative outcome [10]. Modifiable variables in this study included the RAPT score and patient diagnosis of DMII. First, the components that comprise the RAPT score such as use of community supports (e.g. home help, meals-on wheels, district nurse), usage of gait aid, presence of support person at home, and how far the patient can walk, may each be individually optimized [16]. A multidisciplinary healthcare team consisting of community representatives and social workers may be uniquely suited towards helping patients attaining home support and food security [43]. Support of patient enrollment in nutrition assistance programs has also shown great promise in reducing negative health outcomes and would serve to improve the corresponding component of the RAPT score [44]. In conversations with patients, clinicians can emphasize in advance the importance of having a person at home following surgery and give the patient, family, and friends time to make necessary arrangements. Physical therapy can also ensure that the patient can walk as independently as possible and maximize functional mobility prior to surgery [14, 45]. Finally, DMII can be modified to ensure that the patient has the condition under control. Maximum daily glucose variation and peak postoperative glucose have shown to be significant predictors of perioperative wound infection and other medical complications [46]. Thus, hemoglobin A1c should be routinely checked prior to surgery, as it is reasonable to assume that achieving optimal levels (< 7.5) would lead to better outcomes.

Identification of patients at risk of eLOS and discharge to rehabilitation may also assist with a priori discharge planning. Delays in discharge referral can deplete resources for ongoing patients as providers spend more time with newer admissions. Such patients are less frequently re-evaluated, increasing risk of adverse events and medical error [47, 48]. Preoperatively, patients with low RAPT scores, a procedure with a high planned SII, high ASA score, depression or anxiety, and DMII should have prompt administrative teams to set up the rehabilitation referral process in advance.

The strengths of this study include highly granular patient data, rigorous application of inclusion and exclusion criteria, and variables incorporating social support (i.e. RAPT score) in tandem with medical comorbidities. Access to the procedure note enabled manual calculation of SII based on the manual review of the textual information, a feature difficult to attain in studies derived from large administrative datasets. The granular data enabled the SII’s excellent face validity, with higher ranges corresponding to increased OR’s for both outcomes. Moreover, RAPT scores taken directly in the clinical setting and recorded in the patient chart are also unique to this study. Among current literature analyzing risk factors for patients undergoing multi-level fusion for a diagnosis of ASD or lumbar degenerative pathology, this is the first to utilize the RAPT score to capture the patient’s social environment along with demographical, operative, and preoperative clinical variables.

The study should also be interpreted in the context of its limitations. First, utilization of single institution data may lead to results that are less generalizable to other settings, with the tradeoff that single-institution data conferred a higher granularity of data than that of administrative datasets. Lack of socioeconomic variables such as education level and income could be considered a limitation, as they have been proven to provide clinically significant association with postoperative outcomes outside of spine surgery [49]. However, usage of RAPT score enabled the capture of key socioeconomic components (i.e. usage of community programs, presence of support persons), which may explain its high degree of association with eLOS and discharge disposition. Implementation of frailty index, another metric to assess risk for ASD patients, was not feasible in this study and represents a valuable future step [50]. Additionally, even though revision procedures account for many multi-level lumbar/thoracolumbar operations, such procedures were excluded since the diversity of diagnosis included in revisions (i.e. nonunion, junctional pathology, infection) would have created more heterogeneity in the study population. Lastly, inpatient post-operative complications were not assessed, which may have influenced both length of stay and discharge disposition. Despite these limitations, the results of this study should be considered a unique contribution to the growing literature on pre-operative risk assessment of post-operative length of stay and discharge disposition following multi-level instrumented fusions for lumbar degenerative pathology and deformity.

## Conclusion

In this analysis of 355 patients who underwent elective multi-level fusions for ASD and lumbar degenerative pathology, significant variables associated with both eLOS (> 7 days) and discharge to a non-home location (SNF or acute rehabilitation) were higher SII scores and lower RAPT scores. Accurate preoperative assessment of patient risk for eLOS and discharge location has high clinical utility and can enable cost savings, optimization of modifiable risk factors, a priori discharge planning, and management of patient expectations. Given the RAPT score’s high degree of significant association with eLOS and rehabilitation discharge, preoperative optimization of the RAPT’s individual components may provide a useful strategy for decreasing LOS and modifying discharge disposition.

## Data Availability

The data used to support the findings of this study are available from the corresponding author upon request.
